# Assessing the relationship between cardiometabolic diseases and the risk of developing aggressive prostate cancer: a systematic review and meta-analysis

**DOI:** 10.1186/s12885-025-14809-2

**Published:** 2025-10-25

**Authors:** Aurmin J. Amirmokri, Christopher A. Loffredo, Kepher H. Makambi, Nancy A. Dawson

**Affiliations:** 1 Epidemiology Program, Graduate School of Arts and Sciences, Georgetown University, Washington, DC, USA; 2 Department of Oncology, Lombardi Comprehensive Cancer Center, School of Medicine, Georgetown University, Washington, DC, USA; 3 Department of Biostatistics, Bioinformatics, and Biomathematics, School of Medicine, Georgetown University, Washington, DC, USA

**Keywords:** Cohort studies, Diabetes mellitus, Dyslipidemias, Hypertension, Metastasis, Obesity, Prostate cancer, Systematic review and meta-analysis

## Abstract

**Background:**

Prostate cancer is the most prevalent cancer among men within the U.S. and globally, with rising incidence, including advanced-staged disease. Risk factors for aggressive prostate cancer are not well defined. This systematic review and meta-analysis provide an overview of the relationship between cardiometabolic diseases (diabetes, dyslipidemia, obesity, and hypertension) and aggressive prostate cancer.

**Methods:**

Aggressive prostate cancer was defined as disease that has spread or is at high risk of spreading: high-risk or very high-risk localized (T3–T4, Grade Group 4–5), node-positive (N1), or metastatic (M1). Using PRISMA guidelines, a total of 4,830 publications revealed 25 cohort studies of over 974,000 men. Following the systematic review of these prospective studies of men with prostate cancer, R was utilized to run a random effects model, yielding hazard ratios with 95% confidence intervals and generating forest plots with measures of heterogeneity.

**Results:**

Examination of these studies revealed that a positive association exists. Diabetes was associated with a significantly increased risk of aggressive prostate cancer (HR = 1.18; 95% CI: 1.07–1.30; *p* = 0.0008). Obesity also showed a significant association (HR = 1.15; 95% CI: 1.06–1.24; *p* = 0.0006), as did hypertension, though to a lesser degree (HR = 1.07; 95% CI: 1.00–1.14; *p* = 0.04). Dyslipidemia was not significantly associated with aggressive prostate cancer (HR = 1.03; 95% CI: 0.98–1.03; *p* = 0.26).

**Discussion:**

Three of the four cardiometabolic disease components (diabetes, obesity and hypertension) were shown to have statistical significance and offered intriguing evidence on their potential associations with aggressive prostate cancer. Dyslipidemia’s association was not statistically significant, which could be attributed to variations in methods of assessment and differing mechanistic effects. High heterogeneity and limited study availability remain key limitations.

**Conclusion:**

If such associations between cardiometabolic diseases and prostate cancer aggressiveness are shown to be cause and effect, such controllable and treatable conditions can allow oncologists to work alongside primary care physicians to improve patient outcomes and reduce the incidence of aggressive disease. Through the promotion of lifestyle modifications, tighter cardiometabolic control, and targeted interventions, public health efforts might improve prostate cancer outcomes.

**Supplementary Information:**

The online version contains supplementary material available at 10.1186/s12885-025-14809-2.

## Background

Among men today in the United States, prostate cancer (PCa) is the most prevalent malignancy with approximately 1 in 8 men being diagnosed within their lifetime [1]. Approximately 288,300 new cases of PCa arise every year, along with 34,000 deaths [2]. With an aging total population, PCa is affecting more individuals than ever, with an average diagnostic age of 67 years old [1]. Since 2014, the incidence rate for PCa has increased by 3% annually and 5% for advanced-stage PCa [1]. Around 3.1 million men are currently suffering from PCa within the U.S. [1].

Managing aggressive prostate cancer has many challenges: multi-pronged approaches are relied upon to incorporate systematic treatments including chemotherapy, androgen deprivation therapy, androgen receptor pathway inhibitors, PSMA-targeted radioligand therapy [3]. Despite advancements, such cases remain incurable – highlighting a critical need for understanding mechanistic effects involved in its progression.

While localized PCa is frequently treated with therapies such as radiation or surgery, a considerable percentage of patients (40%) still develop metastatic PCa, having a great influence on prognosis and quality of life [2]. Gaps in knowledge persist in the mechanistic pathways that lead to more advanced disease. Localized, regional, and distant stages account for 69%, 13%, and 8% of total PCa cases respectively [2]. Over 100,000 patients are currently living with metastatic PCa cancer within the US [2].

Health disparities are seen as well, with Black Americans experiencing incidence rates 1.7 times higher and death rates 2.1 higher to their White counterparts, and more likely to develop PCa at almost every stage of the disease continuum and within every age group [4]. It is also worth mentioning that Black Americans also experience the highest rates of obesity in the U.S. at 49.6% [5]. Such disparities could be attributed to socioeconomic issues, availability of healthcare access, screening practices, variation in environmental exposures and potentially unexplored genetic predispositions [6].

Cardiometabolic diseases are defined as a cluster of interrelated chronic diseases that collectively increase the risk of cardiovascular disease and metabolic disorders, primarily effecting the heart, blood vessels, blood circulation and metabolism [7]. These are controllable and preventable diseases, with a high prevalence and incidence within the US. The four most prevalent subtypes discussed in this report are obesity, type 2 diabetes mellitus, dyslipidemia and hypertension. To put into perspective the severity of the prevalence of such conditions in the U.S., nearly 136 million Americans suffer from obesity, 105 million from dyslipidemia, 38 million from type 2 diabetes and 120 million from hypertension [8–11].

Obesity is commonly defined as a BMI of 30 kg/m^2^ or greater and is characterized by excessive body fat accumulation [9]. In addition to BMI, waist circumference (WC) is widely used as a clinical marker of central adiposity, with measurements greater than 102 cm in men often indicating abdominal obesity [12]. WC offers complementary information to BMI, as it more specifically reflects visceral fat, which is metabolically active and strongly associated with cardiometabolic risk and cancer outcomes. It is worth noting that obesity can also increase the risk of both dyslipidemia, hyperglycemia and hypertension [13]. Dyslipidemia refers to abnormal lipid levels, which have been defined as a low HDL cholesterol (< 40 mg/dL), high LDL cholesterol (> 130 mg/dL), high total cholesterol (> 240 mg/dL) and/or high levels of triglycerides (> 200 mg/dL); however, definitions may slightly vary based upon the study or data collection method [8]. Type 2 diabetes or hyperglycemia is usually defined as a fasting blood glucose test of 126 mg/dL or greater, and/or hemoglobin A1C testing scores of 6.5% or greater [10]. Hypertension is commonly defined as systolic blood pressure greater than 130 mmHg, or diastolic blood pressure greater than 80 mmHg [11].

This systematic review and meta-analysis focuses on currently published literature while providing insights into potential mechanistic pathways and therapeutic implications. The goal of the study is to access the relationships between the markers of the cardiometabolic diseases—including dyslipidemia, type 2 diabetes, obesity, and hypertension—and how these conditions are associated with the aggressive spread of PCa. The primary hypothesis is that individuals with cardiometabolic diseases are at higher risk of developing aggressive PCa. In turn, a better understanding of these associations, if confirmed in future studies, can promote education on how cardiometabolic diseases increase the risk of advanced PCa, ultimately improving patient outcomes.

## Methods

### Study selection

This systematic review was registered on Open Science Framework (OSF) under the ID FP5QV. We employed Preferred Reporting Items for Systematic reviews and Meta-Analyses (PRISMA) guidelines for the systematic review [14]. PubMed was the primary search database, using the following MeSH terms: ‘Prostatic Neoplasms’, ‘Neoplasm Metastasis’, ‘Disease Progression’, ‘Dyslipidemia’, ‘Obesity’, Diabetes Mellitus, Type 2’, ‘Cohort Studies’, and ‘Hypertension.’ The complete PubMed search strategy, including MeSH terms, free-text keywords, Boolean logic, screening criteria, and search timeframes, is provided in Supplementary Table S1 in accordance with PRISMA guidelines.

Inclusion criteria were peer-reviewed publications from longitudinal cohorts on or after 2015, in English, in humans, full-text original research articles, and investigations reporting associations between aggressive PCa and one or more relevant cardiometabolic diseases. Exclusion criteria were non-human studies, review articles, non-English, published before 2015, non-cohort studies, and studies lacking relevant data on cardiometabolic associations with aggressive PCa.

In addition to PRISMA, we evaluated the included cohort studies using the Strengthening the Reporting of Observational Studies in Epidemiology (STROBE) guidelines to enhance reporting transparency and methodological consistency [15]. We assessed key STROBE domains, such as clarity in study population definitions, exposure classification, outcome ascertainment, follow-up completeness, and handling of confounders. Each study was independently reviewed by two researchers, with discrepancies resolved by a third reviewer.

Prior meta-analyses related to prostate cancer risk factors were reviewed for contextual background. However, none of the individual studies included in these prior meta-analyses were used for data extraction or synthesis in the current analysis. Only primary, independently screened studies identified through our systematic search strategy were included. References to prior meta-analyses serve to contrast differences in study designs, exposure definitions, and analytic approaches compared to the methodology employed in this study. A full list of referenced meta-analyses is provided in the Supplementary Table S4.

**Fig. 1:**
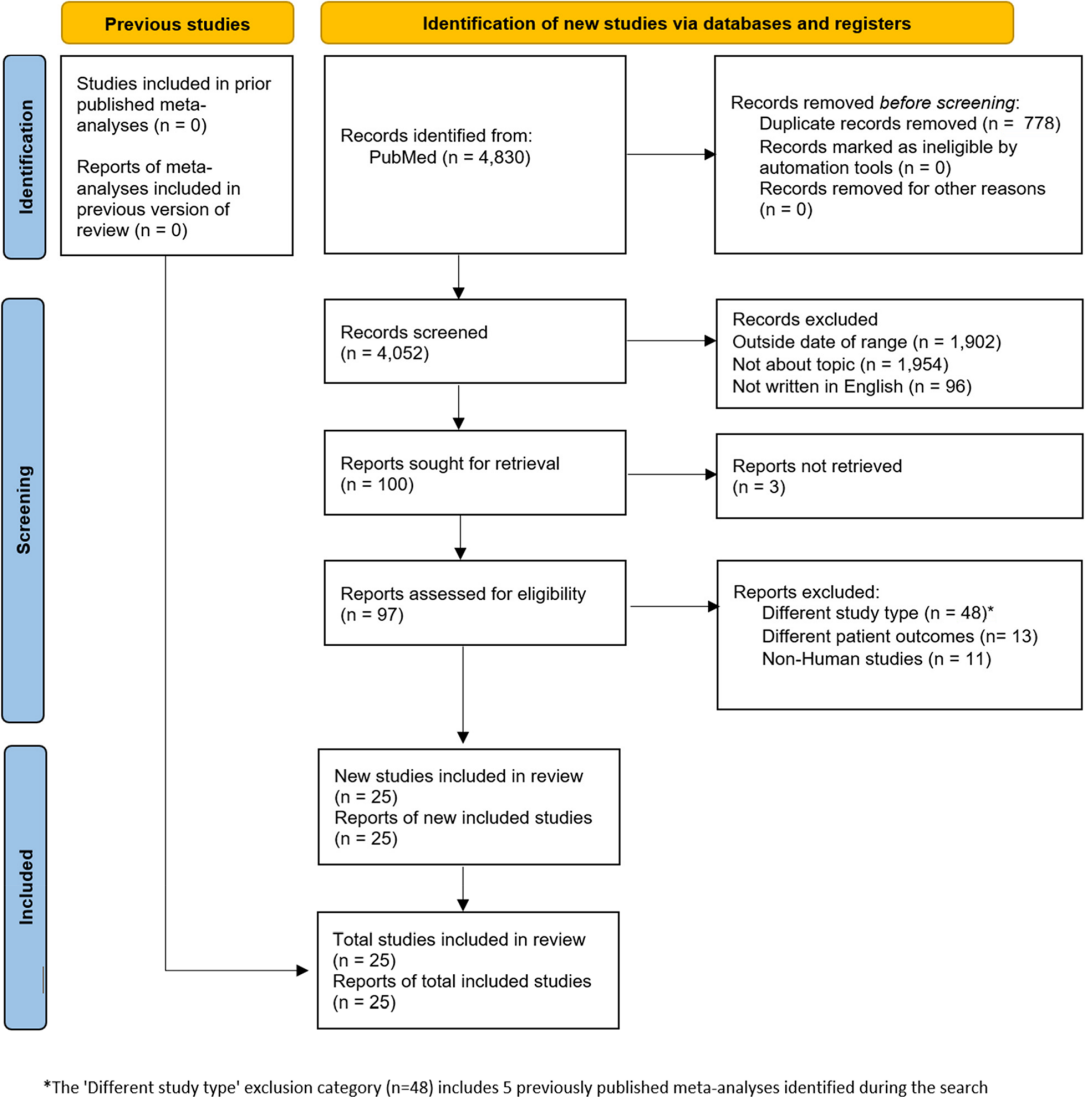
Prisma flow diagram

The PRISMA diagram (Fig. 1) shows the initial number of records identified in PubMed, removal of duplicate reports, irrelevance screening, outside of date range, non-English, differing study types, review articles, and non-human studies. An initial 4,830 records were identified. Duplicate records were removed (*n* = 778), resulting in 4,052 records screened, and thereafter records that were outside of the time span (*n* = 1,902), not about the topic (*n* = 1,954) and not written in English (*n* = 96) were excluded. A total of 97 reports resulted from the above exclusions and were further assessed for eligibility, with 48 excluded due to being a different study type (non-prospective designs or meta-analyses), 13 for different patient outcomes, and 11 for focusing on non-human models – thus leading to 25 studies included in the review.

While 25 individual studies were included in the final synthesis [16–40], a total of 41 distinct data points were extracted, as several studies reported outcomes separately for different cardiometabolic diseases. Across the included studies, prostate cancer staging and grading were highly consistent: the majority employed formal TNM (Tumor, Node, Metastasis) classification, and all studies incorporated Gleason scoring to assess tumor grade. Gleason scores were predominantly high, with most studies including men with scores of 8 or greater, although a small number included patients with scores of 6 or 7, as detailed in the systematic review results. The Gleason score is determined by summing the two most predominant histological patterns of glandular differentiation observed microscopically, each graded from 1 (well-differentiated) to 5 (poorly differentiated), resulting in a composite score ranging from 2 to 10, with higher scores indicating more aggressive disease [41]. Because of the consistency in these clinical definitions, we defined “aggressive prostate cancer” in this study as cancer that has spread or is at high risk of spreading, including high-risk and very high-risk localized disease (T3, T4, Grade Group 4 or 5), node-positive regional disease (N1), and metastatic disease (M1).

Bias assessment in cohort studies included evaluation of participant selection, exposure and outcome measurement accuracy, cohort comparability, and handling of missing data. Risk of bias for each included study was formally evaluated using the Newcastle–Ottawa Scale (NOS), assessing selection, comparability, and outcome domains; with detailed NOS evaluations presented as Supplementary Table S2. Each study was reviewed independently by two reviewers, with discrepancies resolved by consensus. Sensitivity analyses were performed by adjusting key parameters such as inclusion criteria or statistical methods to assess the robustness of the synthesized results. Publication bias due to missing results were evaluated by assessing funnel plot asymmetry.

### Methods of assessment for comorbidities

There were various methods of diagnosis in these studies. For diabetes, very few included only self-reported status, while majority documented the disease from prior medical history, and glucose scores greater than or equal to 126 mg/dL and HbA1c values which are equal or greater than 8.0. Obesity was classified in these studies as a BMI of 30 kg/m^2^ +; however, Asian studies classified it as 25 kg/m^2^ or greater. Moreover, WC of 120 cm or greater has been defined as an additional diagnostic measure for obesity. Dyslipidemia had several different classifications, some which focused on elevated total cholesterol (defined as 188 mg/dL or greater) and/or low HDL cholesterol (observed as 40 mg/dL or less). Hypertension had a few self-reported as well; however, other studies defined hypertension as systolic pressure being greater than 130 mg/dL or diastolic blood pressure being greater than 80 mg/dL.

### Statistical analysis

Statistical analyses were performed using R version 4.3.3 to execute a random effects model and produced summary hazard ratios (HRs) with 95% confidence intervals (CIs). Forest plots were constructed to provide a visual representation of the heterogeneity among the results.

To assess heterogeneity among studies, the I^2^-statistic was used along with Cochran’s Q-statistic. Funnel plots were generated to assess publication bias, heterogeneity, and study precision.

## Results

### Overall association between cardiometabolic disease and aggressive PCa

Table S1 shows the results of the systematic review, with 41 effect estimates shown with confidence intervals, publication year, sample size, age and country origin. Observing that many risk estimates appear > 1.0, with confidence intervals excluding 1.0 as well, there does appear to be a positive overall association between cardiometabolic diseases and aggressive PCa (Table 1). A total sample size of 974,743 individuals were included in this systematic review, with a median age of 65.5 years old. Average period of follow-up for the PCa patients at baseline was 9.8 years (Range: 2—28 years). Approximately 40% of the studies came from various locations in the U.S., however, various countries in Europe and Asia were included as well.

**Table 1:** Summary of pooled hazard ratios and heterogeneity estimates for cardiometabolic conditions associated with aggressive PCa outcomes

Cardiometabolic Condition	No. of Studies	Pooled HR (95% CI)	Heterogeneity (I^2^)	*P*-value
Diabetes	10	1.18 (1.07–1.30)	53%	0.0008
Dyslipidemia	10	1.03 (0.98–1.03)	76%	0.26
Obesity	16	1.15 (1.06–1.24)	66%	0.0006
Hypertension	5	1.07 (1.00–1.14)	51%	0.04

Subgroup analyses were performed to explore sources of heterogeneity. Stratification by sample size (large [*n* > 10,000] vs. small [*n* < 10,000] studies) revealed quite similar HRs across groups at 1.10 (95% CI: 1.05–1.15, *p* < 0.0001) and 1.07 (95% CI: 1.02–1.12, *p* = 0.0044) respectively shown in Supplementary Figure S1. Follow-up time had also been categorically stratified (≤ 5 years, 5–10 years, > 10 years), observing quite similar HRs as well, 1.13 (95% CI: 1.04–1.23, *p* = 0.0029), 1.08 (95% CI: 1.04–1.13,* p* = 0.0004), 1.16 (95% CI: 1.05–1.29,* p* = 0.0039) respectively, indicating little to no difference across follow-up time as visualized in Supplementary Figure S2. Furthermore, stratification of the association between aggressive PCa and the number of cardiometabolic disease(s) present in the study (Supplementary Figure S3) revealed that studies with 2 or more reported cardiometabolic disease revealed a slightly higher HR of 1.11 (95% CI: 1.05–1.17, *p* < 0.0001) as opposed to 1 reported cardiometabolic disease at 1.06 (95% CI: 1.02–1.11, *p* = 0.0044) – indicating that studies with more than 1 reported cardiometabolic disease may have a greater overall influence on effect estimates.

### Diabetes and aggressive PCa

#### Summary

Diabetes had the strongest association with aggressive PCa among the conditions examined, although high heterogeneity was observed across studies.

**Fig. 2:**
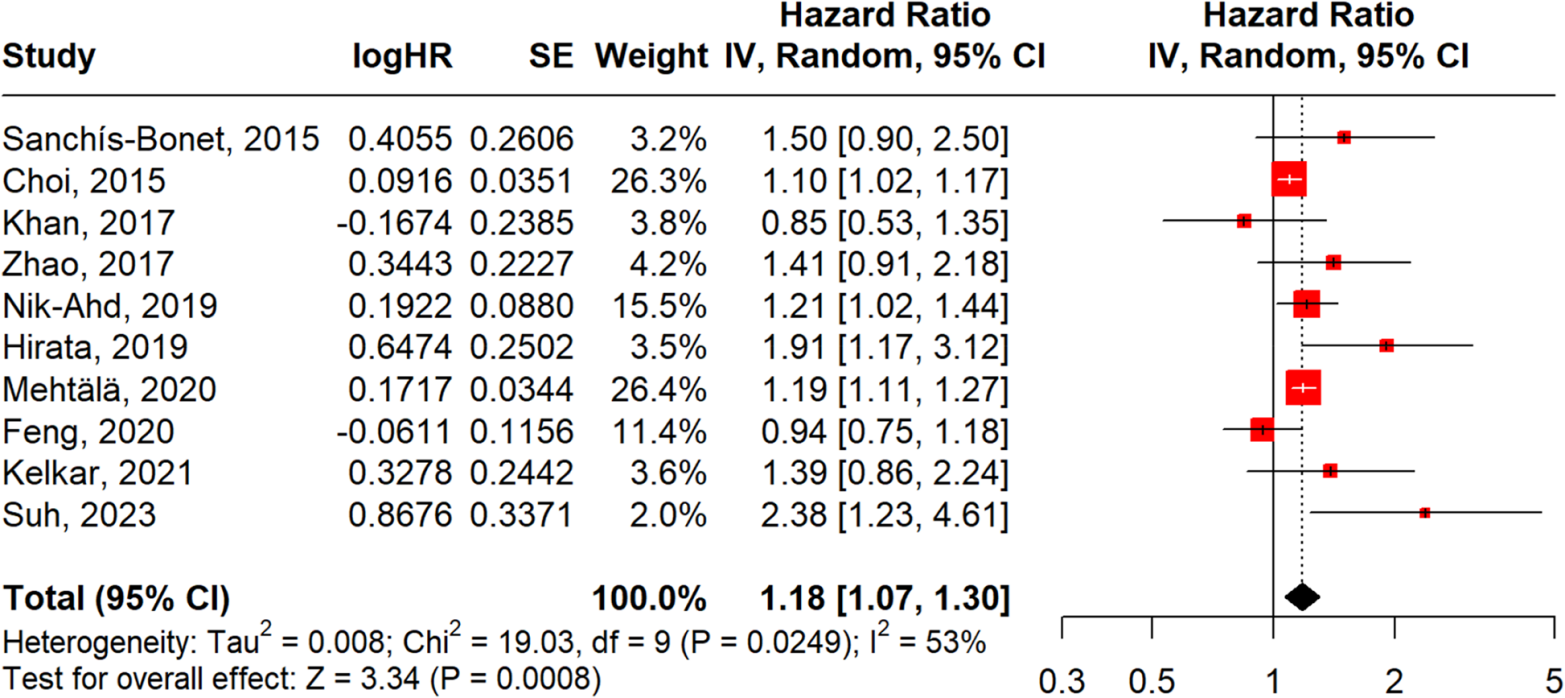
Forest plot, association between diabetes and aggressive PCa

**Fig. 3:**
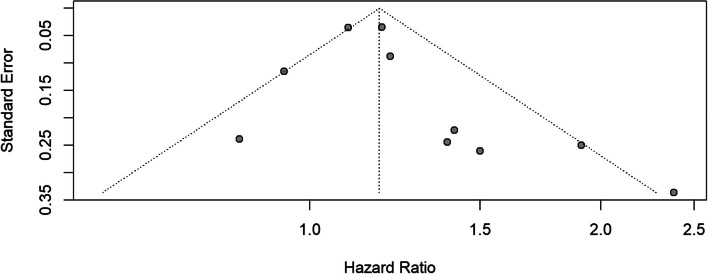
Funnel plot, diabetes and aggressive PCa

The forest plot in Fig. 2 displays results from 10 studies examining diabetes and aggressive PCa. The pooled hazard ratio was 1.18 (95% CI: 1.07–1.30; *p* = 0.0008), indicating a 18% increased risk of aggressive PCa. Heterogeneity was considerable, with I^2^ = 53% and Cochran’s Q = 19.03, df = 9, *p* = 0.0249. These results suggest a consistent direction of effect, albeit with variation in study-level estimates. The corresponding funnel plot (Fig. 3) suggests potential publication bias and imprecision in some studies.

### Dyslipidemia and aggressive PCa

#### Summary

No statistically significant association was observed between dyslipidemia and aggressive PCa.

**Fig. 4:**
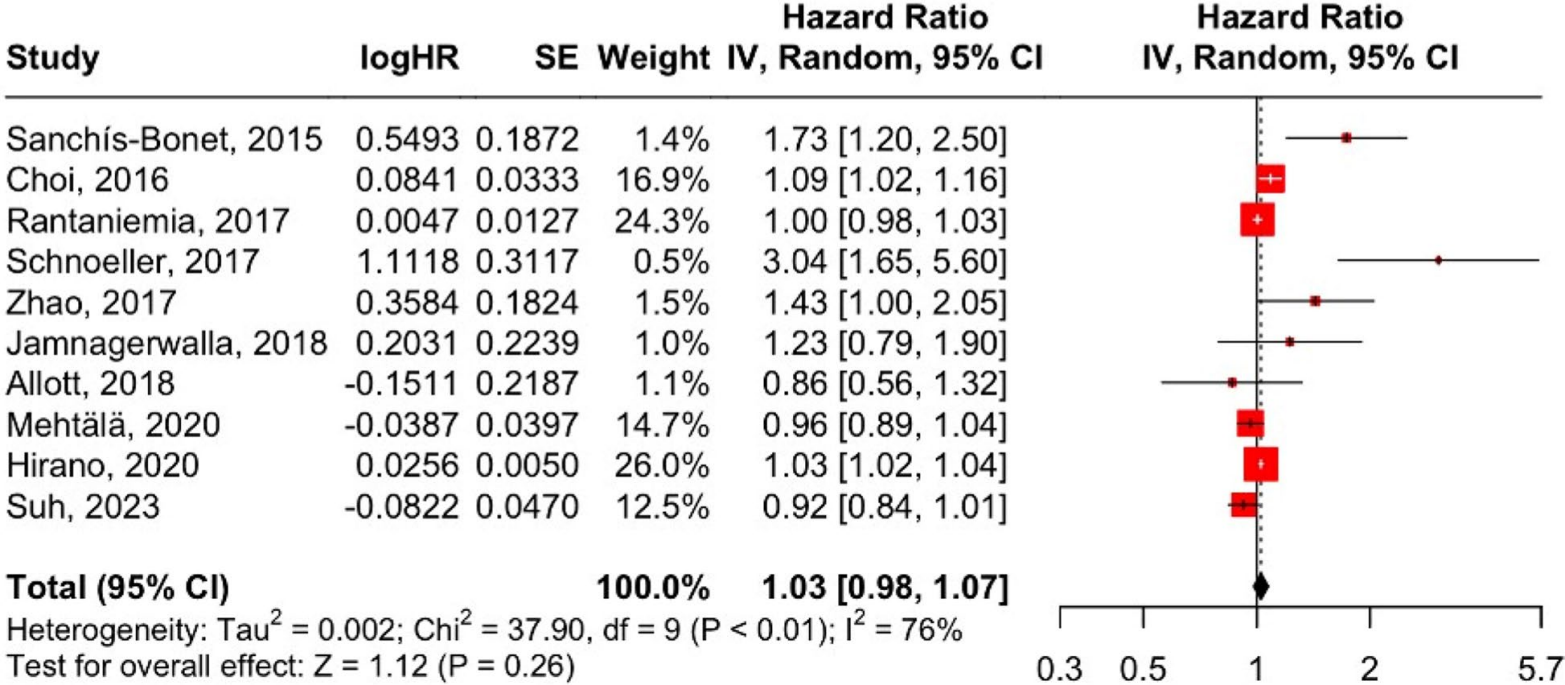
Forest plot, association between dyslipidemia and aggressive PCa

**Fig. 5:**
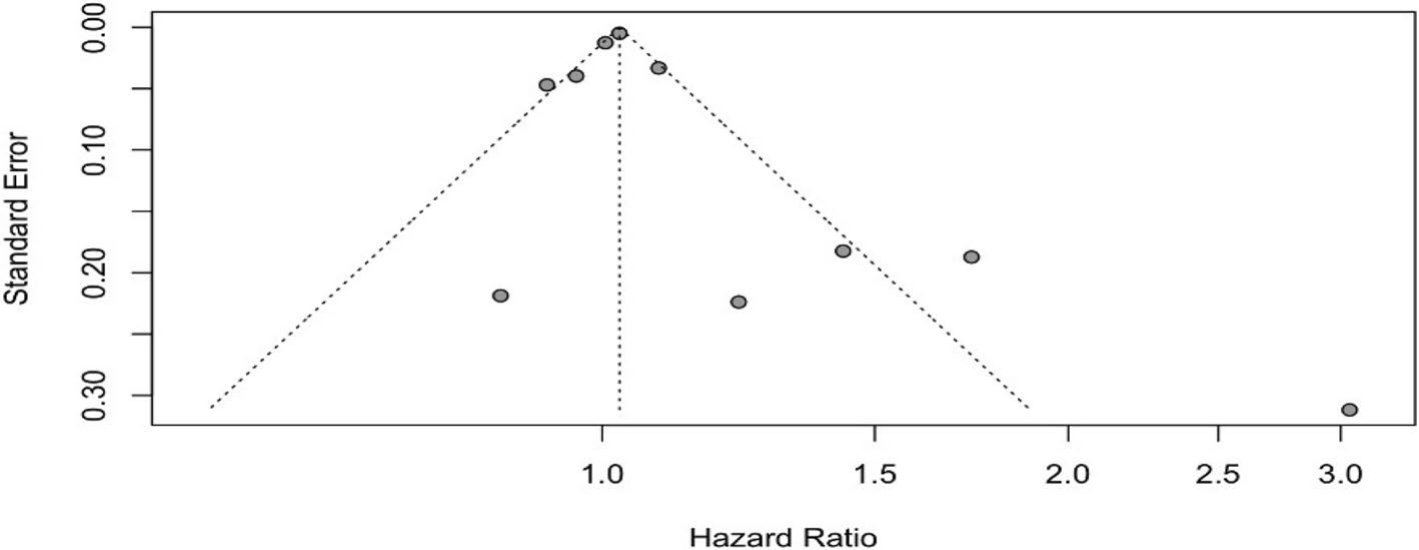
Funnel plot, dyslipidemia and aggressive PCa

As illustrated in Fig. 4, the pooled HR from 10 studies was 1.03 (95% CI: 0.98–1.03; *p* = 0.26). This was the only exposure that did not achieve statistical significance and whose confidence interval included 1. High heterogeneity was again observed (I^2^ = 76%, Cochran’s Q = 37.90, df = 9, *p* < 0.01), and the funnel plot in Fig. 5 shows clustering at the plot’s apex, suggesting precision in most studies, though asymmetry hints at potential bias.

### Obesity and aggressive PCa

#### Summary

Obesity showed a moderate but statistically significant association with increased risk of aggressive PCa.

**Fig. 6:**
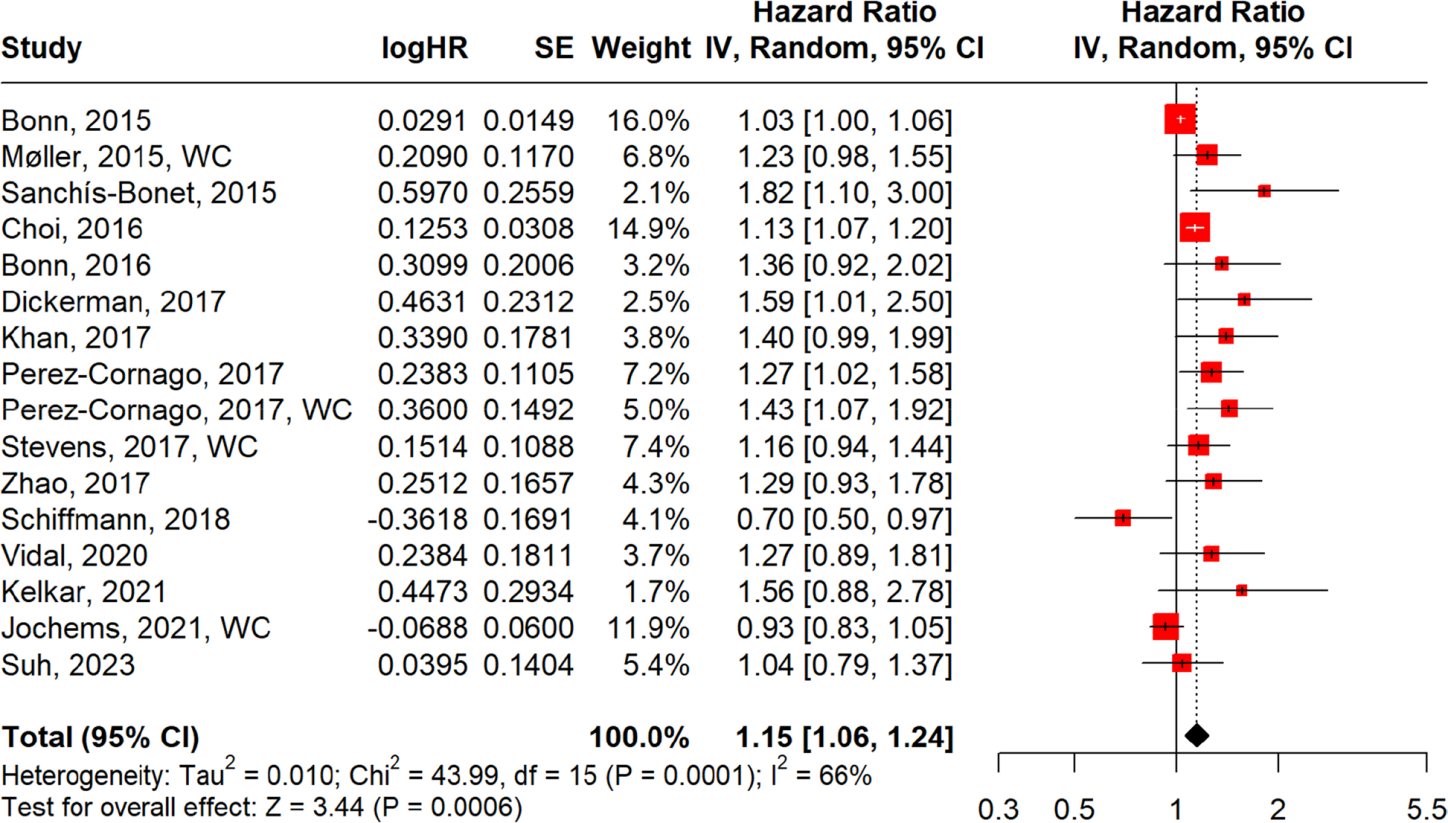
Forest plot, association between obesity and aggressive PCa

**Fig. 7:**
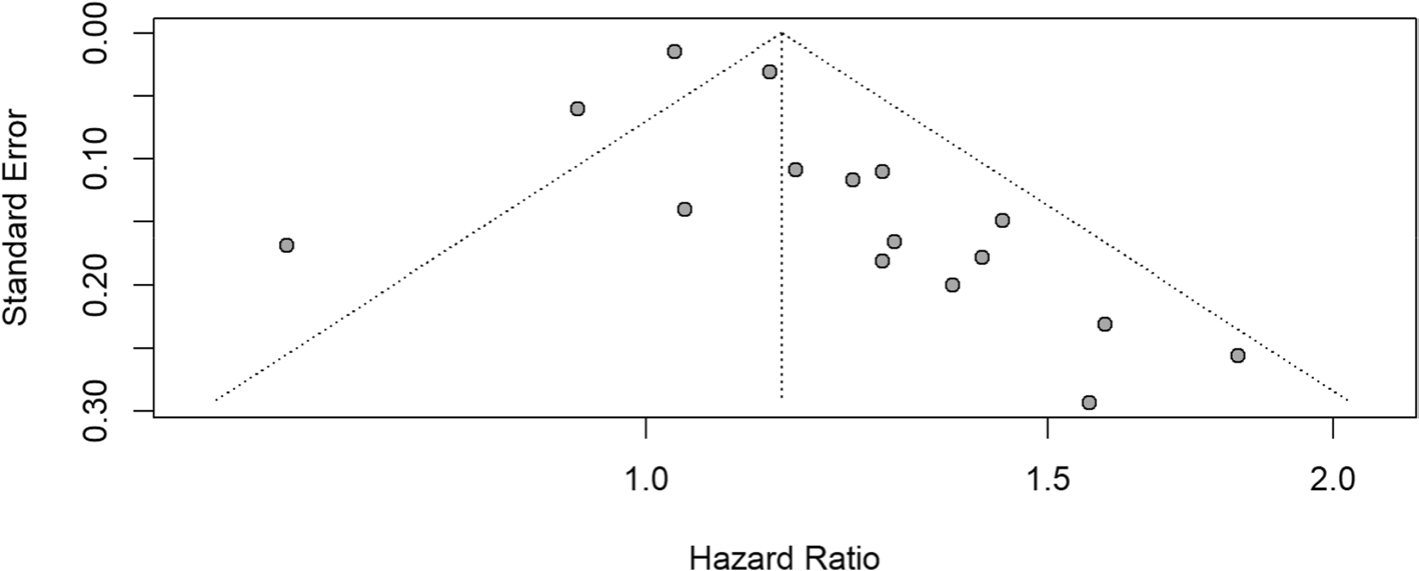
Funnel plot, obesity and aggressive PCa

In Fig. 6, the pooled hazard ratio from 16 studies was 1.15 (95% CI: 1.06–1.24; *p* = 0.0006). This suggests that men with obesity had a 15% increased risk of developing aggressive PCa. Heterogeneity was moderate (I^2^ = 66%, Cochran’s Q = 43.99, df = 15, *p* = 0.0001). The funnel plot (Fig. 7) reveals moderate asymmetry and some dispersion of data points, indicating potential publication bias and variability in effect sizes.

**Fig. 8:**
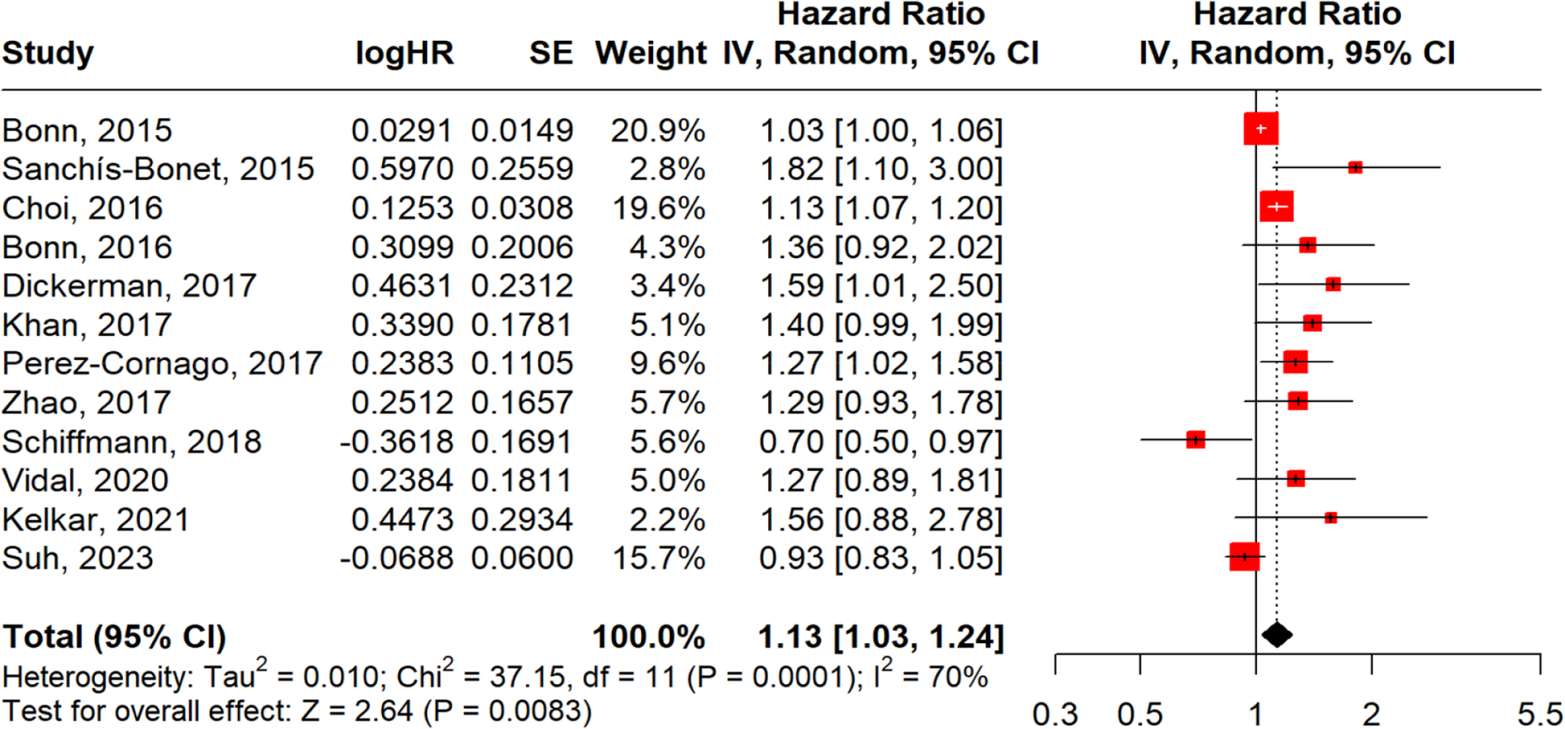
Forest plot, association between obesity BMI and aggressive PCa

**Fig. 9:**
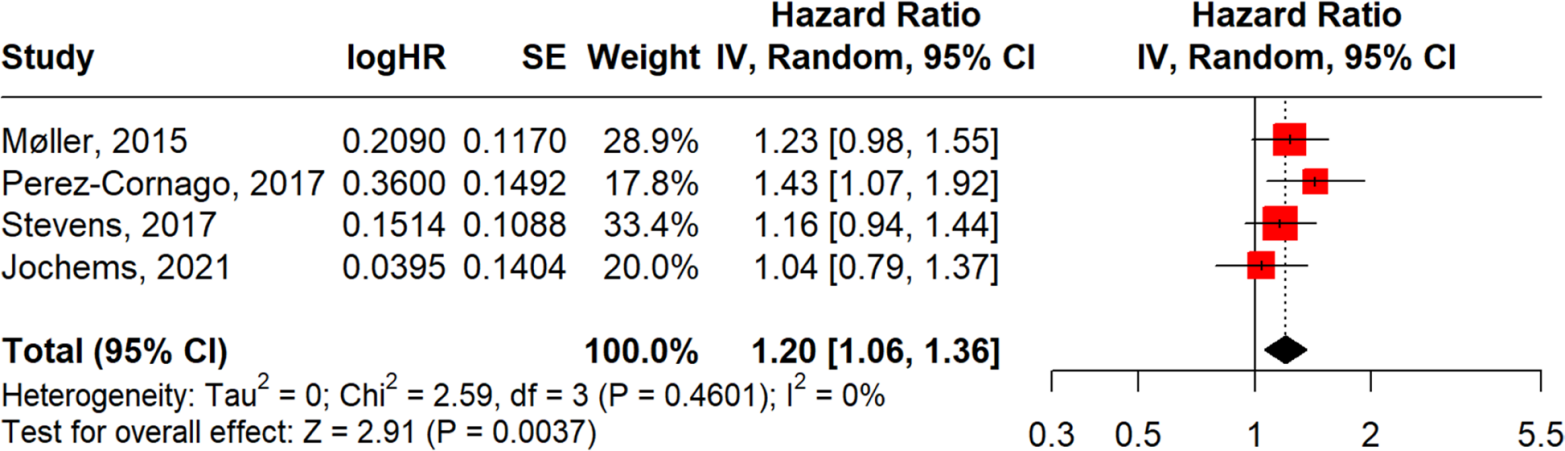
Forest plot, association between obesity WC and aggressive PCa

In subgroup analyses, obesity defined by BMI (Fig. 8) (*n* = 12) yielded a pooled HR of 1.13 (95% CI: 1.03–1.24; *p* = 0.0083; I^2^ = 70%), while obesity defined by WC (Fig. 9) (*n* = 4) showed a slightly stronger association (HR = 1.20, 95% CI: 1.06–1.36; *p* = 0.0037; I^2^ = 0%), though the smaller number of WC studies warrants cautious interpretation.

### Hypertension and aggressive PCa

#### Summary

Hypertension demonstrated a borderline significant association with aggressive PCa, with moderate heterogeneity.

**Fig. 10:**
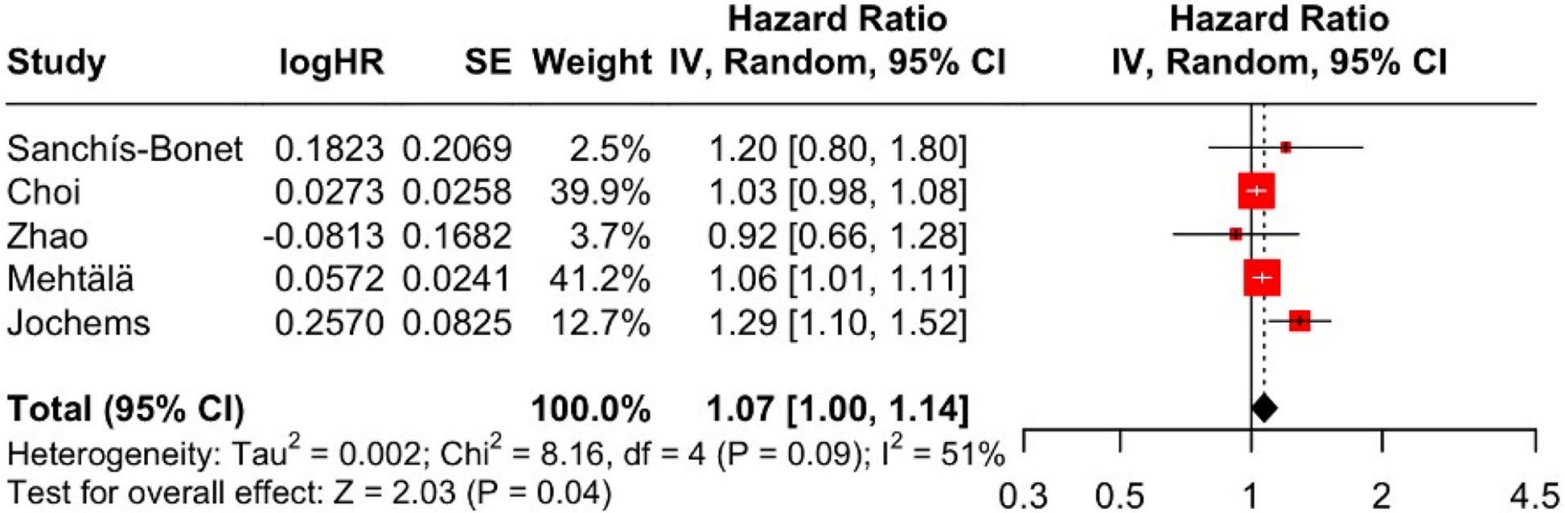
Forest plot, association between hypertension and aggressive PCa

**Fig. 11:**
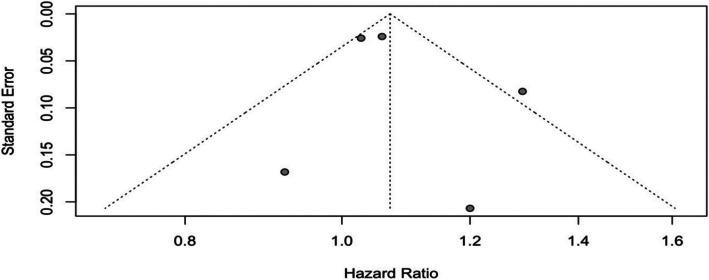
Funnel plot, hypertension and aggressive PCa

As shown in Fig. 10, the pooled analysis from five studies produced an HR of 1.07 (95% CI: 1.00–1.14), with the lower bound of the confidence interval touching the null value. Heterogeneity was moderate (I^2^ = 51%, Cochran’s Q = 8.16, df = 4, *p* = 0.0859). The funnel plot in Fig. 11 shows wide dispersion among data points, reflecting both imprecision and possible reporting bias in smaller studies.

## Discussion

### Summary of findings

To the best of our knowledge, this study provides the first comprehensive quantitative synthesis indicating a potential positive association between pre-existing cardiometabolic diseases and the future development of aggressive PCa. Looking further into the specific types of underlying conditions, diabetes exhibited the strongest association among all cardiometabolic conditions analyzed, with individuals with diabetes facing an 18% higher risk of developing aggressive PCa compared to non-diabetic counterparts. This finding stands out as the most pronounced risk elevation observed in the study, highlighting diabetes as a particularly important comorbidity that may deserve special attention in prostate cancer risk stratification and management efforts. Obesity followed closely, associated with a 15% increased risk of metastasis, reinforcing the growing recognition of adiposity as a driver of aggressive prostate cancer phenotypes [12, 42]. Hypertension was associated with a 7% increased risk of metastasis; however, this finding should be interpreted with caution given that the lower bound of the confidence interval touched the null value of 1.00, suggesting borderline statistical significance. Additionally, moderate heterogeneity (I^2^ = 51%) was observed, likely reflecting differences across studies in the definition and measurement of hypertension, participant characteristics (such as age, race, and disease severity), and inconsistent adjustment for potential confounders including obesity, smoking, and antihypertensive medication use. As suggested by prior research [43], differential effects of antihypertensive drug classes on prostate cancer progression may hold potential for influencing outcomes, although further studies are needed to establish these relationships more definitively. Potential mechanisms linking hypertension to metastasis, including inflammation, angiogenesis, and endothelial dysfunction, have been proposed [44, 45]. In contrast, the association between dyslipidemia and aggressive PCa was not statistically significant. It is notable that dyslipidemia was measured variably across studies—using indicators such as high LDL cholesterol, low HDL cholesterol, elevated triglycerides, or high total cholesterol—which may have contributed to inconsistency. Moreover, distinct biological mechanisms, such as the unique effects of lipid metabolism dysregulation on cellular pathways like Akt and mTOR, may further explain why dyslipidemia differs mechanistically from diabetes, hypertension, and obesity [46, 47]. The lack of a significant association underscores that not all cardiometabolic components contribute equally to aggressive PCa. This distinction is critical, as it highlights which comorbidities—specifically diabetes, obesity, and to a lesser extent hypertension—may warrant closer clinical surveillance or mechanistic investigation in the context of prostate cancer management.

Despite some modest effect sizes and observed heterogeneity, these findings carry meaningful clinical implications, particularly given the high global prevalence of cardiometabolic diseases among men at risk for prostate cancer with an estimated 3.3 million PCa survivors in the U.S. alone [1, 2]. Future studies with more uniform definitions of exposures and more detailed adjustment for key confounders, including pharmacologic treatments, are warranted to refine these associations and guide multidisciplinary strategies for risk stratification and intervention.

A comparison to prior meta-analyses helps contextualize the present findings. A meta-analysis conducted by the *American Association for Cancer Research* examined blood cholesterol levels and prostate cancer risk, finding no significant associations between total cholesterol, LDL, or HDL cholesterol and prostate cancer incidence or mortality [48]. These findings corroborate our result that dyslipidemia was not significantly associated with aggressive prostate cancer; however, our study specifically focused on advanced disease progression rather than overall incidence or mortality. A separate meta-analysis published by *SpringerPlus* in 2016, investigated the impact of diabetes mellitus on prostate cancer mortality, revealing a 29% increased risk of death among diabetic men, supporting our observation that diabetes is an important predictor of more aggressive prostate cancer outcomes [49]. Additionally, a systematic review and meta-analysis using the UK Biobank demonstrated that increased adiposity, particularly waist circumference and waist-to-hip ratio, was significantly associated with higher prostate cancer mortality, consistent with our finding that central obesity may be more predictive of aggressive disease than BMI alone [50]. Similarly, a dose–response meta-analysis found a positive association between higher BMI and prostate cancer mortality, reinforcing the relationship between general obesity and poor survival outcomes [51]. Furthermore, a meta-analysis identified hypertension as a modest but significant risk factor for prostate cancer, although their focus was on incidence rather than aggression, highlighting the novelty of our focus on disease progression [52]. Collectively, while prior meta-analyses established important links between cardiometabolic conditions and prostate cancer outcomes, our study is unique in its exclusive emphasis on aggressive prostate cancer and its integrated evaluation of multiple cardiometabolic risk factors, with Supplementary Table S4 providing a structured comparison of these differences.

### Mechanistic effects

Several cardiometabolic diseases may contribute to aggressive prostate cancer through overlapping biological mechanisms including chronic inflammation, immune cell activation, hormonal imbalances, and metabolic dysregulation.

Chronic inflammation is a recognized biological driver of cancer progression and metastasis, including in PCa. Persistent low-grade inflammation disrupts tissue homeostasis, facilitates DNA damage, and modulates the tumor microenvironment, all of which contribute to tumor progression and dissemination. In particular, inflammation of the vascular endothelium fosters a permissive environment for tumor invasion and angiogenesis — key steps in metastasis [47, 53, 54]. This is notably exacerbated in individuals with obesity, where excess adipose tissue acts as a potent source of pro-inflammatory cytokines such as IL-6, TNF-α, and leptin, which have been shown to enhance tumor cell proliferation, migration, and immune evasion [47, 55, 56].

Importantly, the normal prostate gland expresses high levels of insulin receptors, making it particularly responsive to metabolic changes [42]. In type 2 diabetes mellitus, hyperinsulinemia and elevated insulin-like growth factor 1 (IGF-1) levels drive activation of the PI3K/Akt/mTOR pathway, enhancing cell survival and proliferation [57]. These hormonal shifts also suppress immune surveillance via increased cortisol and adrenal steroid synthesis, facilitating immune evasion and tumor progression [57]. Additionally, insulin resistance further intensifies systemic inflammation, creating a feedback loop that promotes tumorigenic processes [58, 59]. PCa cells have been shown to overexpress insulin receptors, and exposure to diabetogenic insulin and glucose concentrations in vitro enhances their migratory, adhesive, and proliferative capacity [60].

Obesity, especially when induced by a high-fat diet, influences metastatic PCa through hormonal, immunologic, and metabolic pathways. Excess adiposity increases aromatase activity, leading to elevated systemic estrogen levels, which interact with estrogen receptors (ERs) frequently expressed in advanced prostate tumors [42, 55]. This promotes tumor cell proliferation and cross-talk between ER and AR signaling pathways [55]. Obesity also drives infiltration of macrophages into periprostatic adipose tissue, where they secrete IL-6. This cytokine has a dual role—facilitating both inflammatory signaling and androgen receptor (AR) activation, contributing directly to PCa cell proliferation, epithelial–mesenchymal transition, and metastasis [46]. In clinical and experimental studies, elevated IL-6 levels correlate with more aggressive disease and higher mortality in PCa patients [61]. Furthermore, AR overexpression in prostate tumors from diabetic individuals suggests that metabolic stress augments hormone sensitivity and metastatic potential [62]. Notably, WC—a measure of central adiposity—may offer a more precise link to metastatic risk than BMI alone. WC reflects visceral fat, which is metabolically more active than subcutaneous fat and secretes pro-tumorigenic cytokines including leptin, TNF-α, and IL-6, all of which promote a pro-metastatic tumor microenvironment [55]. Elevated WC has been independently associated with advanced-stage PCa and higher mortality, potentially through paracrine signaling that facilitates tumor invasiveness and systemic endocrine disruption [55]. These data suggest that regional fat distribution may be a key mechanistic driver of disease progression, and not just total body mass.

There is strong mechanistic overlap between IL-6 signaling, type 2 diabetes, and obesity, particularly through the activation of STAT3 and AR pathways. Elevated systemic IL-6, commonly observed in both obese and diabetic individuals, has been linked to high-grade PCa and increased androgen receptor sensitivity [46, 54, 55]. These shared inflammatory and hormonal axes provide a robust explanation for our observed associations between metabolic dysregulation and aggressive PCa.

In contrast, the mechanisms linking dyslipidemia to aggressive PCa are less inflammatory and more centered on lipid signaling and cholesterol metabolism. Dyslipidemia alters membrane lipid raft composition, affecting receptor clustering and intracellular signaling [47]. Elevated serum cholesterol facilitates de novo steroidogenesis and enhances AR activation, thus sustaining PCa growth in androgen-depleted conditions [46, 47]. Activated Akt, a kinase essential to prostate cancer growth and progression, has been strongly associated with hypercholesterolemia, PC3 cell proliferation, and increased intratumoral androgen production [63]. These effects underscore the role of cholesterol as more than a passive metabolic factor—it is a key modulator of prostate cancer cell behavior. While inflammation is not the central mechanism in dyslipidemia, its metabolic effects may interact with other cardiometabolic conditions—particularly obesity and diabetes—to exacerbate androgen signaling, tumor cell migration, and metabolic adaptation within the tumor microenvironment [64]. While the association between dyslipidemia and aggressive PCa was not statistically significant in our meta-analysis, this likely reflects the heterogeneity of underlying mechanisms rather than the absence of biological relevance. These findings emphasize the need for disease-specific mechanistic research, as not all cardiometabolic comorbidities contribute equally to cancer aggressiveness.

Hypertension contributes to metastatic potential through mechanical and oxidative stress. Repeated high-pressure states result in vascular remodeling, activating enzymes like matrix metalloproteinase-2 (MMP-2), which degrade the extracellular matrix (ECM) and facilitate cancer cell invasion [44, 45]. Furthermore, endothelial dysfunction, a hallmark of chronic hypertension, promotes angiogenesis, inflammation, and loss of vascular integrity—key steps for tumor cell intravasation and extravasation during metastasis [45]. Studies consistently report upregulated MMP-2 expression and neovascularization in hypertensive individuals, supporting the mechanistic plausibility of this association. Angiogenesis is a common mechanistic denominator in many of these pathways: the formation of new blood vessels not only supports tumor growth, but also provides a route for prostate cancer cells to spread to distant tissues [45].

The present findings reinforce the hypothesis that cardiometabolic dysfunction, particularly diabetes and obesity, are major drivers of prostate cancer aggressiveness. Diabetes was associated with an 18% increased risk of aggressive PCa, while obesity contributed a 15% increased risk, underscoring their clinical relevance. Mechanistically, both conditions converge on shared pathways such as chronic inflammation, hyperinsulinemia, immune suppression, and activation of pro-metastatic signaling cascades including PI3K/Akt/mTOR [65–67]. The role of vascular dysfunction, driven by metabolic syndrome components, further enhances tumor invasion and metastatic potential, as supported by PSA variability and disease severity in hypertensive patients [68]. Notably, Tanaka and Node (2019) emphasized that cardiometabolic syndrome is not merely a comorbidity but an active facilitator of prostate cancer progression, a view strongly aligned with our results [69]. Collectively, these data support an emerging paradigm where integrated metabolic control may be critical not only for cardiovascular outcomes but also for mitigating prostate cancer aggressiveness.

### Strengths and limitations

The major strengths of this study are its objectivity and rigor. Regarding objectivity, following standardized procedures not only minimized the influence of personal biases but aided in validity for the findings through consistent data collection. Such a method encourages repeatability and openness, as every stage of the review process was methodically recorded and reported. Data analyses were conducted equitably and without preference for any specific result. As for the rigor of the study, a clear research design that adhered to PRISMA guidelines was used, and explicit inclusion and exclusion criteria were applied to each and every study that was reviewed. Risk of bias was assessed using the Newcastle–Ottawa Scale, and results are included in the supplementary material as Supplementary Table S2.

On the other hand, this systematic review and meta-analysis is limited by the scarcity and heterogeneity of studies, in turn affecting the robustness and reliability of the findings. The high levels of heterogeneity observed in this study suggest that differences in study methods, populations, outcome definitions, and exposure assessments may have influenced the consistency of reported associations. Most included studies assessed cardiometabolic risk factors through clinical examination, biomarker data, or registry coding. However, two studies relied on self-reported diabetes status, which may introduce recall or misclassification bias. Importantly, the average follow-up period across all studies was 9.8 years, which supports a temporal relationship between cardiometabolic exposures and prostate cancer outcomes and helps mitigate concerns regarding reverse causality. The long lag time also suggests that measured risk factors likely preceded diagnosis in most cases. Additionally, the average cohort size was 38,990 participants, providing sufficient power across studies, although a few included relatively small subsets of patients with advanced or metastatic disease, which may impact the precision of effect estimates. Stratification by study size and follow-up time had also been performed to further assess heterogeneity. The overall findings remained consistent across large and small studies, reducing concerns about small-study bias. A modest gradient in HRs was observed across follow-up time categories, which may reflect latency in prostate cancer progression. However, due to limited availability of retrospective studies and non-clinical exposure definitions, we did not perform subgroup analyses for study design or exposure source.

Additionally, the literature search for this meta-analysis was conducted exclusively through PubMed. While PubMed is a comprehensive and widely-used biomedical database, restricting the search to a single source may have resulted in the omission of relevant studies indexed only in other databases such as Embase, Scopus, or the Cochrane Library. Although manual reference tracking was performed to identify additional eligible studies, this limitation could affect the overall comprehensiveness and generalizability of the review. The complete search strategy has been provided in Supplementary Table S1 for transparency and reproducibility.

Visual inspection of funnel plots revealed moderate asymmetry in several analyses, particularly for obesity and diabetes, suggesting a potential publication bias favoring studies with statistically significant findings. This asymmetry raises concerns about selective reporting and the possible underrepresentation of null or inverse results in the literature. As a result, the strength of some pooled estimates may be inflated, which impacts the generalizability and interpretability of these associations. While formal statistical tests for publication bias were limited due to the number of studies in some subgroups, the patterns observed underscore the need for greater transparency and pre-registration of observational studies in this field.

Another limitation to consider is detection bias, particularly among obese individuals. Higher body mass has been associated with hemodilution of serum PSA levels, which may delay diagnosis and result in underrepresentation of early-stage cases. This could create the appearance of stronger associations between obesity and advanced disease, even when no such biological link exists. Furthermore, collider stratification bias is a potential concern. Since cardiometabolic conditions like diabetes or obesity may influence both the risk of developing prostate cancer and the probability of detection, restricting the analysis to individuals with diagnosed cancer may introduce spurious associations. These types of bias are difficult to correct for in meta-analytic frameworks, but they warrant caution when interpreting associations as causal.

In addition, several of the original studies reported hazard ratios with confidence intervals that crossed 1, indicating that these individual results could not exclude the possibility of no statistically significant difference between the exposed and control groups. This adds further uncertainty to the synthesized estimates, particularly in cases where the pooled result may be influenced by a few larger or more extreme studies. A major limitation is that there were too few studies (*n* = 2) that stratified by race to draw any meaningful conclusions regarding differential associations with aggressive PCa, despite general scientific awareness of the disparities in PCa incidence and outcomes suffered by Black men compared to Whites.

Finally, we acknowledge the potential impact of residual confounding and survival bias. Although multivariable-adjusted estimates were prioritized where available, unmeasured or inconsistently reported variables—such as socioeconomic status, dietary intake, physical activity, and access to healthcare—could influence both the presence of cardiometabolic disease and prostate cancer progression, thereby introducing bias. Additionally, survival bias may be relevant, as individuals with severe cardiometabolic disease may experience premature mortality from non-cancer causes, potentially before metastatic progression is diagnosed. As discussed by Dilixiati et al. [70], such bias can obscure or attenuate the true associations between exposure and cancer outcomes, particularly in older or high-risk populations.

### Public health implications and future research

The possible conjunction of diabetes, hypertension, and obesity as contributors to aggressive PCa carries profound public health implications, resonating across clinical practice, research endeavors, and the fields of oncology and cardiology. Should these associations be corroborated in future studies, clinicians may gain greater awareness of the metastatic risks associated with cardiometabolic comorbidities and implement more tailored surveillance strategies for affected patients. Multidisciplinary care teams—including oncologists, urologists, obesity specialists, cardiologists, and primary care physicians—may be well-positioned to personalize treatment and monitoring regimens based on these risk profiles.

In particular, therapies aimed at improving cardiometabolic health have generated interest as potential adjuncts to standard prostate cancer treatment. While agents such as metformin have shown biologically plausible mechanisms—such as modulation of insulin signaling, mTOR inhibition, and anti-inflammatory effects—clinical evidence to date remains inconclusive. For example, the STAMPEDE trial found no survival advantage with the addition of metformin in men with metastatic prostate cancer, although it did reduce metabolic toxicity from androgen deprivation therapy [71]. Similarly, the MAST trial, evaluating metformin in low-risk prostate cancer during active surveillance, found no effect on disease progression [72]. A broader lifestyle intervention study also found no consistent evidence for dietary or metabolic agents significantly modifying prostate cancer outcomes, though improvements in quality of life and comorbidities were observed [73]. These findings suggest that while cardiometabolic therapies may not offer direct oncologic benefit in all settings, they could still play a supportive role in managing treatment-related side effects and comorbidity burden.

In the future, targeted surveillance and therapies could be informed through clarifying the underlying biological processes relating obesity, hypertension, and diabetes to the progression of PCa. Additional longitudinal cohort studies would be essential to validate the current literature and could also test for the effects of cardiometabolic therapies (i.e. pharmaceutical treatments and lifestyle changes) on cancer progression. Multidisciplinary cooperation between urologists, cardiologists, and oncologists, to name a few, can allow for comprehensive treatment plans in PCa patients with cardiometabolic comorbidities. Conducting research in medical systems such as the VA, that care for large numbers of African American men with prostate cancer would also contribute to a better understanding of the underlying disparities in incidence and mortality, especially given the high prevalence of cardiometabolic diseases in this group.

Although racial disparities are well-documented in prostate cancer outcomes, particularly among Black men, only two studies in our review stratified their findings by race [17, 40]. This limited our ability to evaluate differential associations. We highlight this as an important limitation and call for future cohort studies to routinely report and stratify findings by racial or ethnic groups to better understand population-specific risk patterns.

## Conclusion

There are positive associations of obesity, diabetes and hypertension with aggressive PCa in the current literature. If such associations are shown to be cause and effect, oncologists can work with primary care physicians to determine if tighter control of cardiometabolic disease can improve cancer outcomes through promotion of surveillance for metastatic disease, lifestyle modifications, tighter cardiometabolic control, weight loss programs and targeted interventions.

## Supplementary Information

Supplementary Material 1

## Data Availability

The dataset generated and analyzed in the study are available on the Zenedo repository (https://doi.org/10.5281/zenodo.15287990] (https://doi.org/10.5281/zenodo.15287990). All data used in this meta-analysis and systematic review were included from previously published studies. These studies are publicly accessible and can be found in the respective journals listed in the references section of this manuscript. The detailed search strategy, including the databases searched and the keywords used, is provided in the methods under Supplementary Figure 1.

## Abbreviations

PCa: Prostate cancer
US: United States
BMI: Body mass index
PRISMA: Preferred reporting items for systematic reviews and meta-analyses
STROBE: Strengthening the reporting of observational studies in epidemiology
PSA: Prostate-specific antigen
MeSH: Medical subject headings
HR: Hazard ratio
RCT: Randomized controlled trial
PSMA: Prostate-specific membrane antigen
WC: Waist circumference
