# Supplementary material for: Assessing the relationship between cardiometabolic diseases and the risk of developing aggressive prostate cancer: a systematic review and meta-analysis
Source: BMC Cancer. 2025 Oct 25;25:1645. doi: 10.1186/s12885-025-14809-2 (PMC12553227; doi:10.1186/s12885-025-14809-2)
Supplement: Supplementary file 1 — Supplementary Material 1 [file 12885_2025_14809_MOESM1_ESM.docx]

**Supplementary Materials**

**Supplementary Table S1. Complete Search Strategy and Screening Process**

1. **Search Strategy Details**

| **Item** | **Details** |
| --- | --- |
| **Database Searched** | PubMed |
| **Date Range** | January 1, 2015 – Feb 1, 2024 |
| **Search Fields** | Title, Abstract, MeSH terms |
| **Language Filter** | English |
| **Population Filter** | Humans |
| **Search Terms and Strategy** | ("prostate cancer"[MeSH Terms] OR "prostatic neoplasms"[MeSH Terms] OR "PC"[Title/Abstract] OR "PCM"[Title/Abstract] OR "prostate carcinoma"[Title/Abstract] OR "mPCa"[Title/Abstract]) AND ("metastasis"[Title/Abstract] OR "progression"[Title/Abstract] OR "development"[Title/Abstract] OR "aggressiveness"[Title/Abstract] OR "high-grade"[Title/Abstract] OR "advanced"[Title/Abstract]) AND ("diabetes mellitus"[MeSH Terms] OR "diabetes"[Title/Abstract] OR "diabetes mellitus type 2"[Title/Abstract] OR "obesity"[MeSH Terms] OR "obese"[Title/Abstract] OR "body mass index"[Title/Abstract] OR "overweight"[Title/Abstract] OR "adiposity"[Title/Abstract] OR "dyslipidemias"[MeSH Terms] OR "dyslipidemia"[Title/Abstract] OR "hyperlipidemia"[Title/Abstract] OR "lipid metabolism disorders"[Title/Abstract] OR "hypertension"[MeSH Terms] OR "elevated blood pressure"[Title/Abstract]) |
| **Boolean Operators Used** | OR within categories; AND across categories |
| **Additional Filters** | None beyond language and human studies |

**B. Screening and Selection Process**

| **Stage** | **Description** |
| --- | --- |
| **Title and Abstract Screening** | Conducted independently by two reviewers based on relevance to prostate cancer outcomes and cardiometabolic exposures. |
| **Full-Text Review** | Full texts of eligible articles were reviewed to confirm inclusion criteria were met. |
| **Discrepancy Resolution** | Discrepancies were resolved through discussion and consensus. A third reviewer was consulted if needed. |
| **Inclusion Criteria** | Cohort studies (prospective or retrospective) reporting associations between diabetes, obesity, hypertension, or dyslipidemia and prostate cancer outcomes (progression, recurrence, metastasis, mortality) with HRs, ORs, or RRs and 95% CI. Human studies, English language. |
| **Exclusion Criteria** | Reviews, meta-analyses, case reports, editorials, animal studies, studies without sufficient effect size data. |

**Supplementary Figure S1. Forest Plot, Association between Sample Size and Aggressive PCa**

**
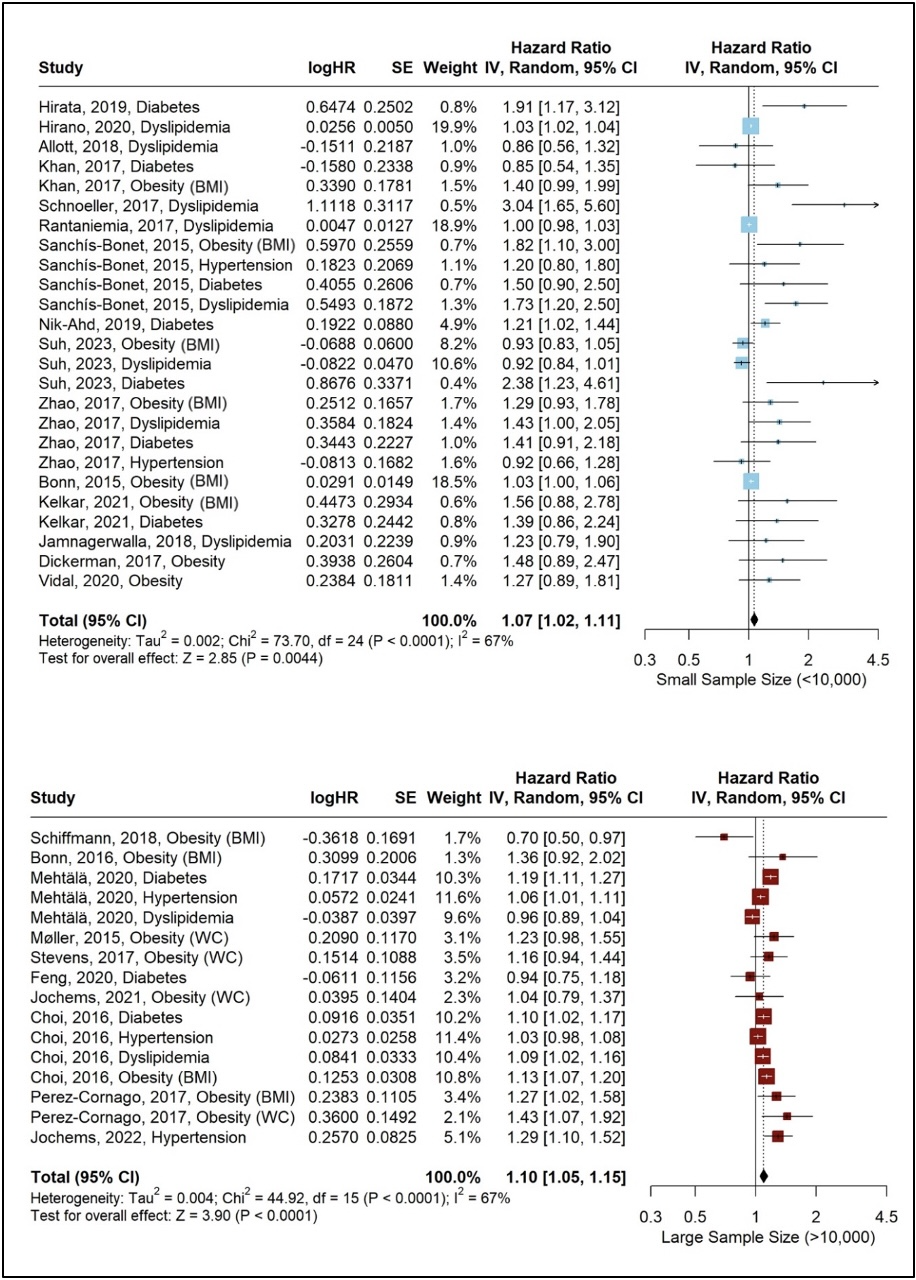
**

**Supplementary Figure S2. Forest Plot, Association in Lengths of Follow-Up and Aggressive PCa**

**
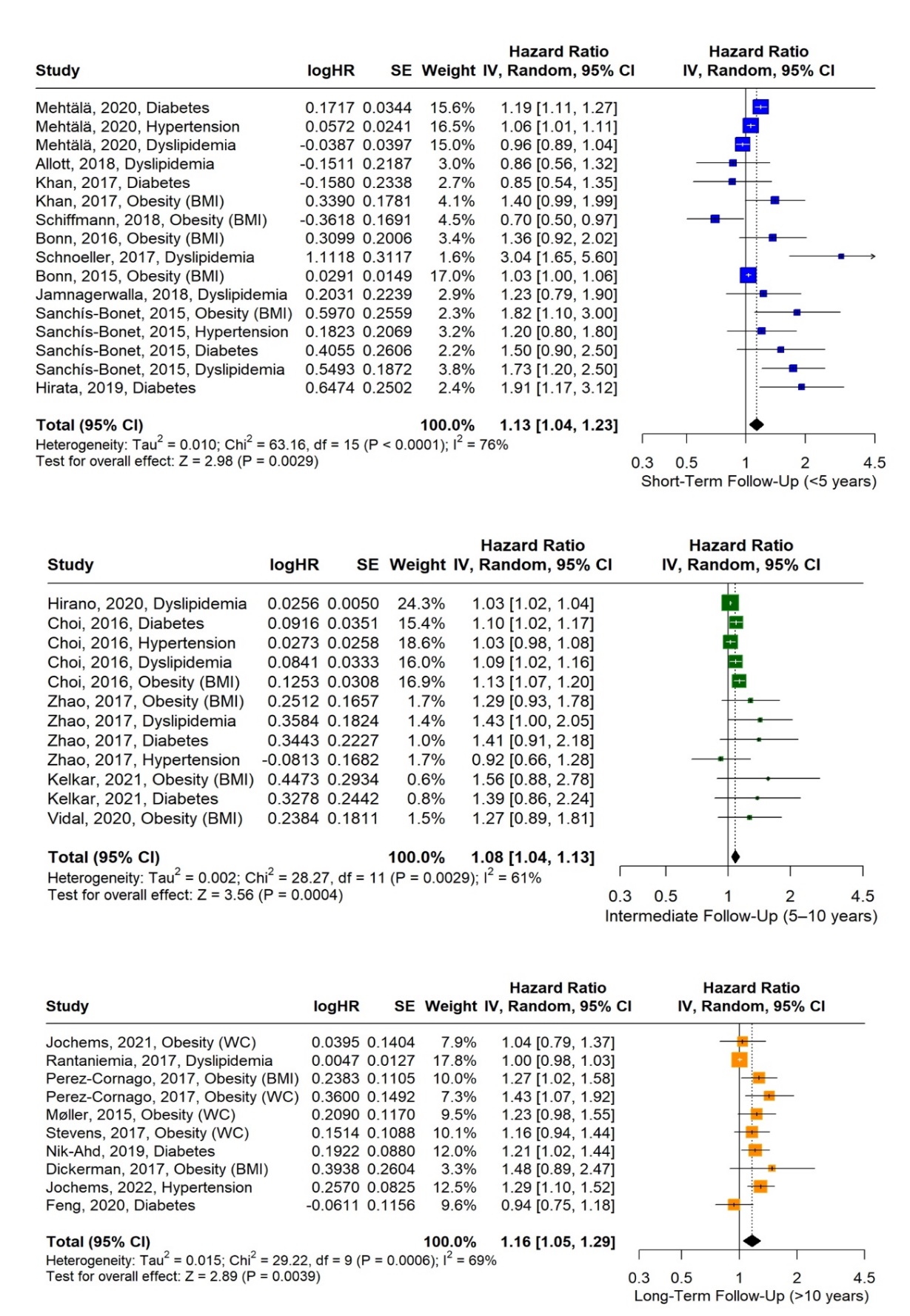
**

**
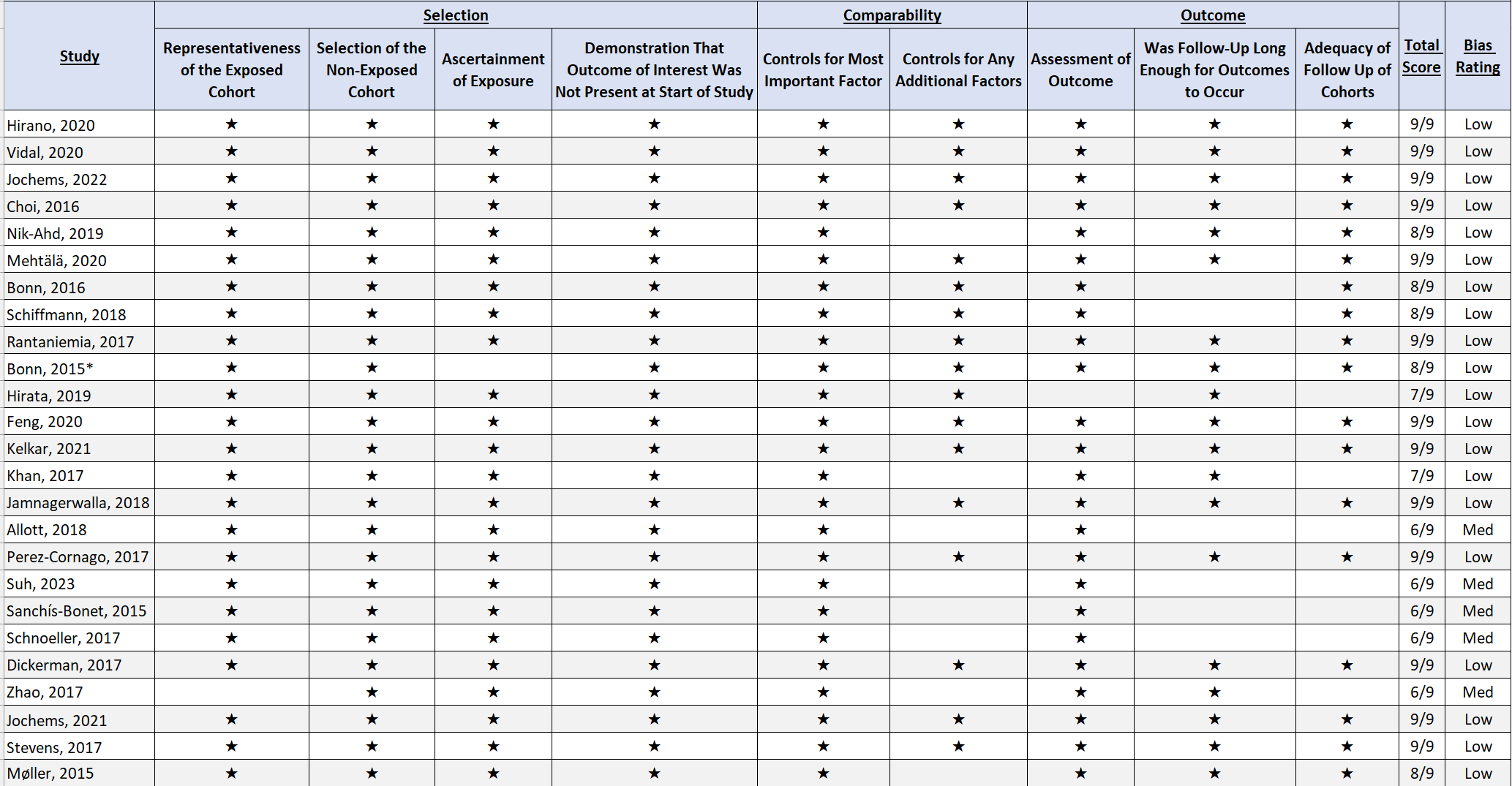
Supplementary Table S2. Risk of Bias Assessment, Newcastle-Ottawa Scale for Cohort Studies**

**Supplementary Table S3. Descriptive Characteristics of Studies Used in Meta-Analysis**

| **Category** | **Author** | **Year** | **Study Type** | **Risk Statistic Measurement** | **Risk Value** | **Lower 95% CI** | **Upper 95% CI** | **Sample Size** | **Number Of Advanced PCa Cases** | **Percent of Cardiometabolic Disease Present** | **Average Age** | **Country** | **Risk of Bias Score** |
| --- | --- | --- | --- | --- | --- | --- | --- | --- | --- | --- | --- | --- | --- |
| Dyslipidemia | Hirano et al. | 2020 | Prospective Cohort | RR | 1.03 | 1.016 | 1.036 | 351 | 108 | 25.07% | 70 | Japan | Low |
| Obesity (BMI) | Vidal et al. | 2020 | Prospective Cohort | HR | 1.27 | 0.89 | 1.81 | 5,929 | 1,934 | 33.78% | 63 | US | Low |
| Hypertension | Jochems et al. | 2022 | Prospective Cohort | HR | 1.29 | 1.1 | 1.52 | 430,472 | 1,854 | 37.40% | 38 | Sweden | Low |
| Diabetes | Choi et al. | 2016 | Prospective Cohort | HR | 1.1 | 1.023 | 1.174 | 139,519 | 7,622 | 11.96% | N/A | South Korea | Low |
| Hypertension |  |  |  |  | 1.03 | 0.977 | 1.081 |  |  | 39.44% |  |  |  |
| Dyslipidemia |  |  |  |  | 1.09 | 1.019 | 1.161 |  |  | 16.03% |  |  |  |
| Obesity (BMI) |  |  |  |  | 1.13 | 1.067 | 1.204 |  |  | 36.16% |  |  |  |
| Diabetes | Nik-Ahd et al. | 2019 | Prospective Cohort | HR | 1.21 | 1.02 | 1.44 | 1,409 | 59 | 100.00% | 62 | US | Low |
| Diabetes | Mehtälä et al. | 2020 | Retrospective Cohort | HR | 1.19 | 1.11 | 1.27 | 15,953 | 15,953 | 9.73% | 73.9 | Sweden | Low |
| Hypertension |  |  |  |  | 1.06 | 1.01 | 1.11 |  |  | 24.04% |  |  |  |
| Dyslipidemia |  |  |  |  | 0.96 | 0.89 | 1.04 |  |  | 6.43% |  |  |  |
| Obesity (BMI) | Bonn et al. | 2016 | Prospective Cohort | HR | 1.36 | 0.92 | 2.02 | 15,827 | 282 | 13.58% | 65 | Sweden | Low |
| Obesity (BMI) | Schiffmann et al. | 2018 | Prospective Cohort | HR | 0.7 | 0.5 | 0.97 | 13,667 | 1,047 | 14.60% | 64 | Germany | Low |
| Dyslipidemia | Rantaniemia et al. | 2017 | Prospective Cohort | HR | 1.01 | 0.98 | 1.03 | 1,314 | ~ 679 | 35.90% | 62 | Finland | Low |
| Obesity (BMI) | Bonn et al. | 2015* | Prospective Cohort | HR | 1.03 | 1 | 1.06 | 4,376 | ~ 810 | 11.00% | 65 | Sweden | Low |
| Diabetes | Hirata et al. | 2019 | Prospective Cohort | HR | 1.94 | 1.17 | 3.12 | 121 | 98 | 22.30% | 72 | Japan | Low |
| Diabetes | Feng et al. | 2020 | Prospective Cohort | HR | 0.94 | 0.75 | 1.18 | 49,392 | 2,513 | 9.80% | 71.3 | US | Low |
| Obesity (BMI) | Kelkar et al. | 2021 | Prospective Cohort | HR | 1.57 | 0.88 | 2.78 | 4,688 | ~ 770 | 33.30% | 62 | US | Low |
| Diabetes |  |  |  |  | 1.38 | 0.86 | 2.24 |  |  | 20.40% |  |  |  |
| Diabetes | Khan et al. | 2017 | Prospective Cohort | HR | 0.86 | 0.54 | 1.35 | 672 | 83 | 17.90% | 62 | US | Low |
| Obesity (BMI) |  |  |  |  | 1.4 | 0.99 | 1.99 |  |  | 38.20% |  |  |  |
| Dyslipidemia | Jamnagerwalla et al. | 2018 | Prospective Cohort | HR | 1.23 | 0.79 | 1.9 | 4,974 | 218 | 5.40% | 63 | US | Low |
| Dyslipidemia | Allott et al. | 2018 | Prospective Cohort | HR | 0.86 | 0.56 | 1.32 | 669 | 250 | 63.50% | 61 | US | Med |
| Obesity (BMI) | Perez-Cornago et al. | 2017 | Prospective Cohort | HR | 1.26 | 1.022 | 1.576 | 141,896 | 1,388 | 15.30% | 68 | Europe | Low |
| Obesity (WC) |  |  |  |  | 1.43 | 1.07 | 1.92 |  |  | 20.00% |  |  |  |
| Obesity (BMI) | Suh et al. | 2023 | Retrospective Cohort | OR | 0.93 | 0.83 | 1.05 | 1,740 | 153 | ~ 1.70% | 66 | South Korea | Med |
| Dyslipidemia |  |  |  |  | 0.92 | 0.84 | 1.01 |  |  | ~ 32.20% |  |  |  |
| Diabetes |  |  |  |  | 2.38 | 1.23 | 4.61 |  |  | 16.30% |  |  |  |
| Obesity (BMI) | Sanchís-Bonet et al. | 2015 | Prospective Cohort | OR | 1.9 | 1.1 | 3 | 1,319 | 252 | 15.00% | 66 | Spain | Med |
| Hypertension |  |  |  |  | 1.2 | 0.8 | 1.8 |  |  | 36.00% |  |  |  |
| Diabetes |  |  |  |  | 1.5 | 0.9 | 2.5 |  |  | 20.00% |  |  |  |
| Dyslipidemia |  |  |  |  | 1.7 | 1.2 | 2.5 |  |  | 34.00% |  |  |  |
| Dyslipidemia | Schnoeller et al. | 2017 | Prospective Cohort | HR | 3.04 | 1.65 | 5.6 | 767 | 358 | 37.40% | 68 | US | Med |
| Obesity (BMI) | Dickerman et al. | 2017 | Prospective Cohort | HR | 1.48 | 0.89 | 2.47 | 5,158 | 371 | 10.90% | 70 | US | Low |
| Obesity (BMI) | Zhao et al. | 2017 | Prospective Cohort | OR | 1.29 | 0.929 | 1.779 | 3,102 | 459 | 37.90% | 69 | China | Med |
| Dyslipidemia |  |  |  |  | 1.43 | 1.001 | 2.046 |  |  | 20.30% |  |  |  |
| Diabetes |  |  |  |  | 1.41 | 0.912 | 2.183 |  |  | 18.80% |  |  |  |
| Hypertension |  |  |  |  | 0.92 | 0.663 | 1.282 |  |  | 35.40% |  |  |  |
| Obesity (WC) | Jochems et al. | 2021 | Prospective Cohort | HR | 1.05 | 0.79 | 1.37 | 58,457 | 483 | 28.40% | 53 | Sweden | Low |
| Obesity (WC) | Stevens et al. | 2017 | Prospective Cohort | RR | 1.16 | 0.94 | 1.44 | 46,094 | 809 | 40.00% | 68 | US | Low |
| Obesity (WC) | Møller et al. | 2015 | Prospective Cohort | HR | 1.23 | 0.98 | 1.55 | 1,813 | 626 | 25.60% | 56 | Denmark | Low |

**
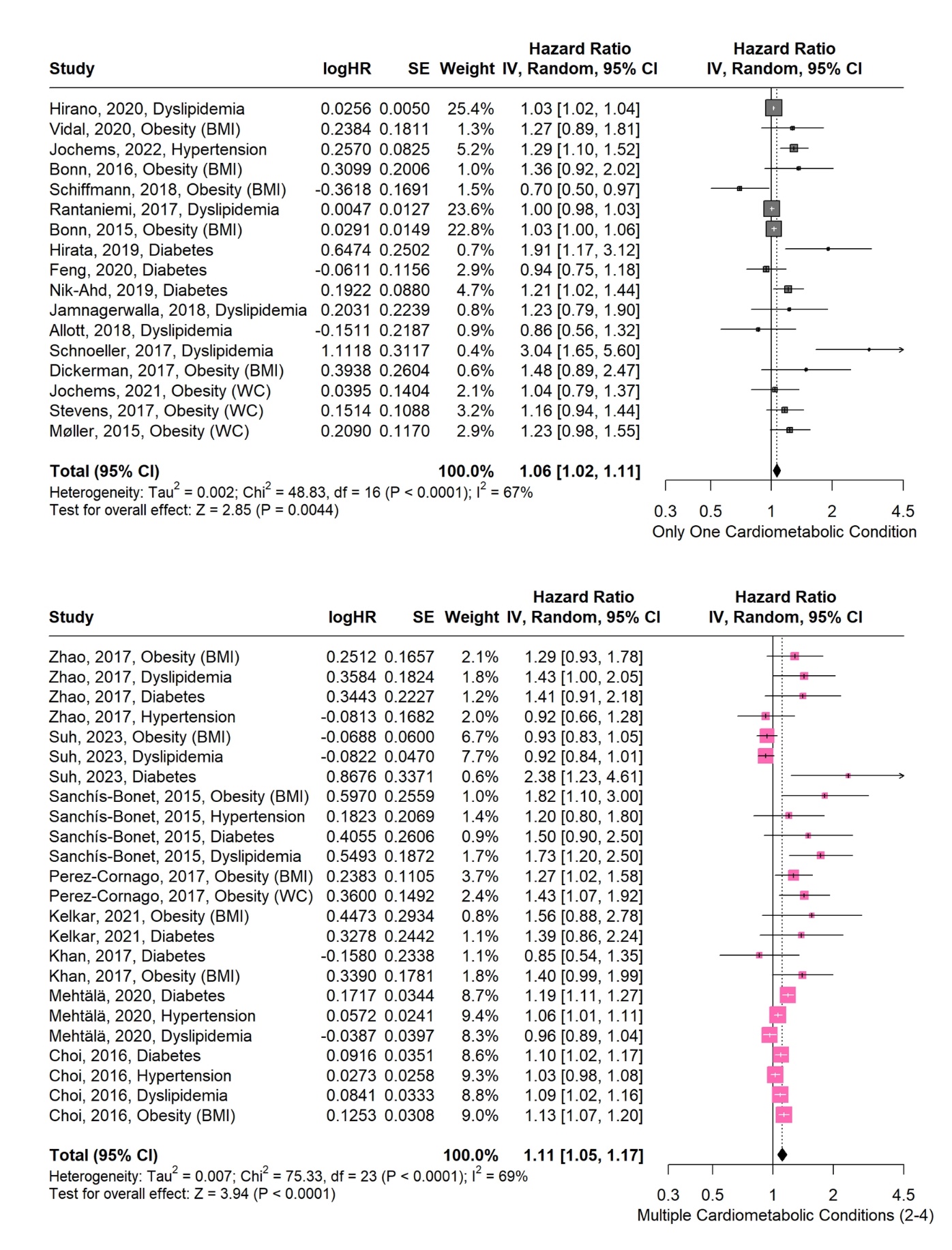
Supplementary Figure S3. Forest Plot, Association of Aggressive PCa with Amount of Cardiometabolic Conditions Present in the Respective Study**

**Supplementary Table S4. Summary of Previously Published Meta-Analyses Reviewed for Context**

| **Reference** | **Focus / Exposure** | **Primary Outcomes** | **Key Differences from Current Study** |
| --- | --- | --- | --- |
| YuPeng, 2015 (*American Association of Cancer Research*) | Blood cholesterol levels | Prostate cancer incidence and mortality | Focused on general PCa risk; did not analyze aggressive or metastatic outcomes or cardiometabolic clustering |
| Lee, 2016 (*SpringerPlus*) | Diabetes mellitus | Prostate cancer-specific mortality | Examined diabetes and mortality; did not assess metastasis-specific risk |
| Perez-Cornago, 2022 (*BMC Medicine*) | Adiposity (waist circumference, waist-to-hip ratio) | Prostate cancer mortality | Mortality-focused, not metastasis-specific; assessed central adiposity but not hypertension, diabetes, dyslipidemia |
| Rivera-Izquierdo, 2021 (*Cancers MDPI*) | Body mass index (BMI) | Prostate cancer mortality | Studied BMI and mortality; did not integrate diabetes, hypertension, or dyslipidemia as exposures |
| Liang, 2016 (*Scientific Reports, naturesearch*) | Hypertension | Prostate cancer risk | General PCa risk only; no focus on progression; hypertension assessed independently |
